# Geometry and Surface Area Optimization in Iron Oxide Nanoparticles for Enhanced Magnetic Properties

**DOI:** 10.1021/acsomega.4c03988

**Published:** 2024-07-18

**Authors:** Alexis Lavín Flores, Nataniel Medina-Berríos, Wenndy Pantoja-Romero, Dariana Berríos Plaza, Kim Kisslinger, Juan Beltran-Huarac, Gerardo Morell, Brad R. Weiner

**Affiliations:** † Molecular Sciences Research Center, University of Puerto Rico, San Juan, Puerto Rico 00926-2614, United States; ‡ Department of Chemistry, University of Puerto Rico, Río Piedras Campus, San Juan, Puerto Rico 00925-2537, United States; § Department of Physics, University of Puerto Rico, Río Piedras Campus, San Juan, Puerto Rico 00925-2537, United States; ∥ Department of Biology, College of Natural Sciences, University of Puerto Rico, Rio Piedras Campus, San Juan, Puerto Rico 00925-2537, United States; ⊥ Center for Functional Nanomaterials, Brookhaven National Laboratory, Upton, New York 11973, United States; # Department of Physics, Howell Science Complex, East Carolina University, Greenville, North Carolina 27858, United States

## Abstract

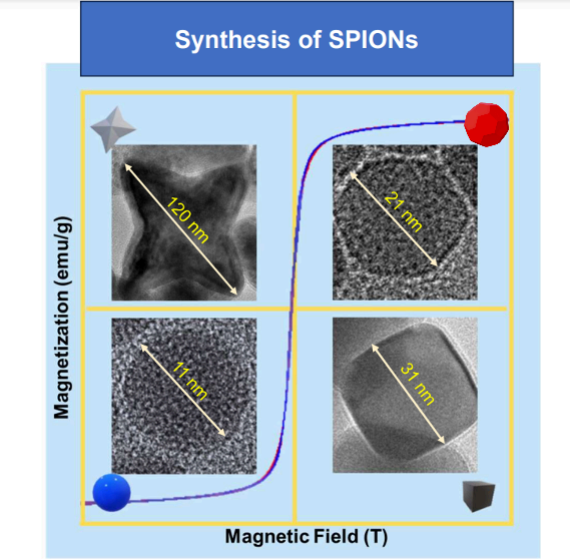

Iron oxide nanoparticles (IONPs) are recognized for their potential in biomedical applications due to their distinctive physicochemical properties. This study investigates the synthesis of IONPs with various geometric morphologies—cubic, star-like, truncated icosahedron, and spherical—via thermal decomposition to enhance their utility in magnetic resonance imaging (MRI) and targeted drug delivery. X-ray diffraction analysis verified the Fe_3_O_4_ phase in all nanoparticles, illustrating the synthesis’s efficacy. Particle morphologies were well-defined, with sizes ranging from 10 to 150 nm, as determined by transmission electron microscopy (TEM) and scanning electron microscopy (SEM). Magnetic evaluations using a vibrating sample magnetometer (VSM-PPMs) demonstrated their superparamagnetic behavior, with larger particles exhibiting greater saturation magnetization. Notably, truncated icosahedron and cubic IONPs showed superior transverse relaxation rates, with r_2_ values of 56.77 s^1^ mM^1^ and 42.67 s^1^ mM^1^, respectively. These results highlight the potential of customizing IONP geometries to optimize their magnetic properties and increase surface area available for functionalization, thereby improving their efficacy for biomedical applications.

## Introduction

Nanostructured systems have been extensively studied for their unique physicochemical properties and potential applications in nanomedicine.^1^ Iron oxide nanoparticles (IONPs) have emerged as a promising tool for various biomedical applications, owing to their biocompatibility and ability to design versatile moieties for a range of applications, e.g. to promote tissue regeneration,^2^ transport of drugs or genes in cell therapy,^3,4^ diagnostic purposes in multimodal molecular imaging,^5^ cancer therapy^6^ and anemia treatment.^7^

Magnetic resonance imaging (MRI) is a preferred clinical diagnostic tool that utilizes the contrast between different tissues to generate high-resolution images of different penetration depth.^8−10^ MRI contrast agents are necessary to enhance the visibility of pathologic tissue by affecting the relaxation times (T_1_ and T_2_) of the resonant protons.^11^ While gadolinium-based contrast agents (GBCAs) have been widely used in clinical diagnostics, they are associated with significant health concerns, including toxicity and allergic reactions.^12,13^ The quest for efficient contrast agents for noninvasive medical imaging has led researchers to explore the unique magnetic properties of IONPs. Their superparamagnetic behavior, tunable magnetic responses, and size-dependent characteristics make them attractive candidates for enhancing the quality and precision of MRI. By modulating the relaxation times of water molecules in biological tissues, these IONPs hold the promise of improving diagnostic accuracy and enabling early disease detection. As an alternative, contrast agents based on IONPs (CAs-IONPs), such as Ferumoxytol, are being explored as an alternative due to their relative stability and lower metal release and deposition.^14^

The biomedical applications of IONPs depend on their surface properties, morphology, size, and magnetization. The surface of IONPs can be coated with various organic or inorganic materials to enhance their selectivity and colloidal stability in biological environments. These coatings can also enable the loading of different drugs or molecules onto the surface of IONPs, which can be delivered to specific targets using magnetic fields.^14^ The morphology and size of IONPs can be controlled by different synthesis methods, which can produce nanoparticles with different shapes, such as spherical,^15^ cubic,^16^ hexagonal^17^ rod-shape,^18^ or flower-like.^19^ The shape and size of IONPs affect their magnetic responsiveness and heating efficiency, which are important for applications such as magnetic resonance imaging (MRI) and magnetic hyperthermia. The magnetization of IONPs is determined by their composition, structure, and size. Higher magnetization can improve the performance and biocompatibility of IONPs for biomedical applications. Therefore, it is essential to optimize and control their tendency to oxidize, while maintaining their paramagnetic and superparamagnetic properties for each specific application of IONPs.^20,21^

Based on quantum-mechanical outer-sphere theory,^22,23^ the T_2_ relaxivity is strongly influenced by the M_s_ value and the effective radius of the superparamagnetic core, which is usually spherical. The relaxivity, r_2,_ can be expressed by the following equation in the motional average regime, where all the nanoparticles are assumed to be spherical.
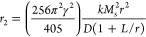
1

In eq 1,^24^ M_s_ and r are the saturation magnetization and the effective radius of the magnetic nanostructure, respectively, D is the water molecule diffusivity, L is the thickness of a nonpenetrable surface coating, and k is the conversion factor (k = V*/CFe), where V* is the volume fraction and CFe is the iron element concentration. Equation 1 shows that a higher r_2_ value can be achieved by increasing either the M_s_ value or the effective magnetic core radius. The M_s_ value is limited by the maximum M_s_ of bulk magnetite, which is about 92 emu g^–1^ at room temperature (emu= electromagnetic unit^25^). Therefore, a more effective way to enhance the T_2_ relaxivity is to increase the effective radius of the magnetic core, which depends largely on the morphology of the nanostructure.^26^

This research explores the geometry-dependent magnetic properties of IONPs and their potential applications in MRI. The crystallographic phases and morphological studies of the synthesized IONPs are examined using X-ray diffraction (XRD) and transmission electron microscopy (TEM) techniques. The optical properties of the IONPs are characterized using Raman spectroscopy and attenuated total reflection Fourier-transform infrared spectroscopy (ATR-FTIR). Additionally, the magnetic properties of the IONPs are analyzed through hysteresis curves measured using a vibrating sample magnetometer (VSM-PPMs) at different temperatures. By comprehensively studying the magnetic and optical properties of IONPs with different geometries, this research aims to provide insights into the correlation between the structural characteristics and the magnetic behavior of IONPs. Such knowledge can contribute to developing tailored IONPs with optimized properties for specific biomedical applications.

## Experimental Section

### Preparation of Iron Oleate Precursor

Iron(III) oleate was synthesized by dissolving iron(III) chloride hexahydrate (FeCl_3_ •6H_2_O, Sigma-Aldrich, 98%) and sodium oleate (97%, TCI) in 60 mL of deionized water (DIW). The mixture was then combined with a solution of 220 mL of ethanol/hexene in a 1:2 ratio. The resulting solution was refluxed at 70 °C for 4 h. The iron(III) oleate product was extracted with a separatory funnel, and washed three times in deionized water. Finally, the hexene solvent was removed by a rotary evaporator.

### Synthesis of IONPs

The thermal decomposition synthesis process was selected due to the versatility and superior control over the shape and morphology of the resulting nanoparticles. Compared to other synthesis methods, thermodecomposition allows for precise manipulation of reaction parameters (Table 1), leading to uniform and monodisperse particles. This method ensures reproducibility and scalability, making it a pertinent choice for producing high-quality nanomaterials. The effectiveness in controlling particle characteristics enhances the applicability of the synthesized nanoparticles in various biomedical and industrial applications.^27,28^

**Table 1:** Synthesis Parameters and Yields for Different Types of Iron Oxide Nanoparticles

nanoparticle type	oleic acid/organic solvent	*T* (°C)	batch time (h)	total initial reactant weight (g)	final product quantity (g)	yield (%)
ST-IONPs	1.7 mL/45 mL (TOA)	340	8	2.52 (iron oleate only)	0.54	24
SQ-IONPs	2.0 mL/45 mL (TOA)	320	2	1.64 (including NaCl)	0.32	20
SP-IONPs	0.22 mL/40 mL 1-Octadecene	320	4	1.6 (iron oleate only)	1.56	35
TI-IONPs	0.22 mL/40 mL (1-Octadecene)	320	2	1.6 (iron oleate only)	0.54	33

### Star-Type Iron Oxide Nanoparticles (ST)

ST-IONPs were prepared by mixing 2.52 g (2.8 mmol) of iron oleate and 1.7 mL (5.4 mmol) of oleic acid with 45 mL of tri-n-octylamine (TOA, 97%, ACROS Organics) in a 250 mL three-neck round flask. The temperature was gradually increased to 340 °C and maintained for 8 h with stirring. Ethanol was added to the final product, followed by centrifugation and discarding of the supernatant. The residue was resuspended via sonication in hexene and washed twice with DIW, using magnetic decantation.

### Cubic Iron Oxide Nanoparticles (SQ)

SQ-IONPs were synthesized by mixing 1.6 g (1.77 mmol) of iron oleate, 2 mL (6.33 mmol) of oleic acid, and 40 mg of NaCl (98%, Sigma) in 45 mL of tri-n-octylamine (TOA, 97%, ACROS Organics in a 250 mL three-neck round-bottom flask. The mixture was refluxed at 320 °C for 2 h under a nitrogen atmosphere. The cleaning process was carried out using the same procedure as the synthesis of ST-IONPs.

### Spherical Iron Oxide Nanoparticles (SP)

SP-IONPs were synthesized using the thermal decomposition method. A mixture of 1.6 g (1.77 mmol) of iron oleate and 220 μL (0.7 mmol) of oleic acid (NF/FCC, Fisher Chemical) dissolved in 40 mL of 1-octadecene (95%, Sigma) was prepared in a three-neck round-bottom flask. The reaction mixture was refluxed at 320 °C for 4 h under a nitrogen atmosphere. The final product was subjected to centrifugation with isopropanol in a 50 mL tube at 10,000 rpm for 30 min. The remaining fraction was washed twice with hexene and separated using magnetic decantation. The remaining fraction was washed two times with hexene and separated using magnetic decantation.

### Truncated Icosahedron Iron Oxide Nanoparticles (TI)

TI-IONPs were synthesized using the thermal decomposition method. A mixture of 1.6 g (1.77 mmol) of iron oleate and 220 μL (0.7 mmol) of oleic acid (NF/FCC, Fisher Chemical) dissolved in 40 mL of 1-octadecene (95%, Sigma) was prepared in a three-neck round-bottom flask. The reaction mixture was refluxed at 320 °C for 2 h under a nitrogen atmosphere. The final product was subjected to centrifugation with isopropanol in a 50 mL tube at 10,000 rpm for 30 min. The remaining fraction was washed twice with hexene and separated using magnetic decantation.

## Results and Discussion

### Crystallographic Phases and Morphological Studies

Higher temperatures and longer reaction times, as seen with star-type IONPs, facilitate anisotropic growth and the formation of complex shapes. Conversely, shorter reaction times and specific temperatures, as with cubic and truncated icosahedron IONPs, favor the stabilization of particular crystallographic planes. Oleic acid acts as a surfactant, selectively binding to specific facets of the nanoparticles. The concentration of oleic acid and the type of solvent (TOA vs 1-octadecene) play crucial roles in determining the final shape.^29−31^

**Figure 1:**
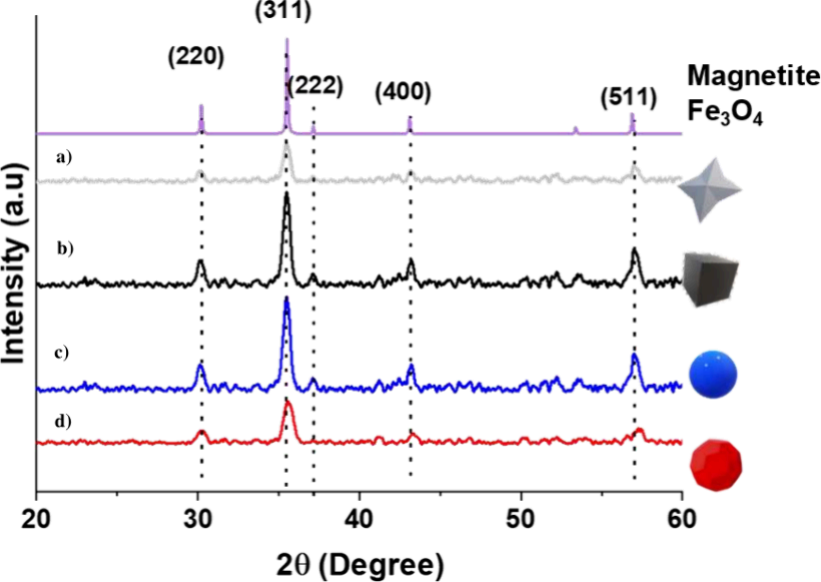
Powder XRD patterns of iron oxide nanoparticles (IONPs) synthesized through the thermal decomposition process: (a) starlike, (b) cubic, (c) spherical and (d) truncated icosahedron.

The X-ray diffraction (XRD) results of the nanomaterial suggest the formation of a magnetite phase with characteristic diffraction peak patterns in (2,2,0), (3,1,1), (2,2,2) confirms the formation of magnetite and suggests that the nanomaterial crystallizes as a spinel on a cubic lattice with the *Fd*3̅*m* space group with Z = 8 (See Figure 1).^32^ In the centrosymmetric description of this space group, the tetrahedral cations occupy the positions (1/8, 1/8, 1/8), the octahedral cations are located at (1/2, 1/2, 1/2), and the oxygen atoms are at (x, x, x), where x is approximately 0.25. To evaluate the crystal phases in the samples, the experimental lattice constant (α) was determined using Bragg’s relationship (eq 2):^33^
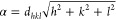
2where (*h k l*) are the Miller indexes and *d*_*hkl*_ are the interplanar spacing obtained from the relationship the wavelength of incident X-rays, angle of incidence and spacing between the crystal lattice planes, expressed as (eq 3)^34^
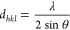
3

The obtained (α) values of the samples varied from 8.373 to8.391 Å (see Table 2), similar to the referenced magnetite bulk material (see Table 3), showing less than 1% error and a cell volume of 587–590 Å^3^^32^ The intensity and width of these diffraction peaks provide valuable information about the crystal structure and purity of the material, the quality of the fit of the experimental data indicates the accuracy and precision of the analysis.^35,36^ The average crystallite size was estimated using the Scherrer eq 4,5)^37,38^ assuming an isotropic model for cubic, spherical, and truncated icosahedron given by
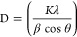
4where D is the average crystallite size, K is a dimensionless shape factor (0.9), β is the full width at half-maximum (fwhm) of the peak, corrected for instrumental broadening and measured in radians, and θ is the Bragg angle, corresponding to the position of the peak maximum. In the context of considering starlike shapes as anisotropic, the modified Scherrer equation was applied as follows:^39^

**Table 2:** Structural Parameters for the Four IONP Geometries (ST, SQ, SP, TI)

	Miller indices									
IONPs				Bragg’s angle 2θ (deg)	*d*-spacing (Å)	lattice constant (Å)	average (Å)	std^32^ (Å)	% error	cell volume (Å^3^)
ST	2	2	0	30.116	2.965	8.386	8.391	8.396	0.07	590.71
	3	1	1	35.522	2.525	8.375				
	2	2	2	37.189	2.416	8.368				
	4	0	0	43.167	2.094	8.376				
	5	1	1	56.567	1.626	8.447				
SQ	2	2	0	30.174	2.959	8.370	8.378	8.396	0.20	588.40
	3	1	1	35.497	2.527	8.381				
	2	2	2	37.155	2.418	8.375				
	4	0	0	43.167	2.094	8.376				
	5	1	1	56.948	1.616	8.395				
SP	2	2	0	30.116	2.965	8.386	8.382	8.396	0.17	588.99
	3	1	1	35.497	2.527	8.381				
	2	2	2	37.114	2.420	8.385				
	4	0	0	43.225	2.091	8.365				
	5	1	1	56.948	1.616	8.395				
TI	2	2	0	30.116	2.965	8.386	8.373	8.396	0.28	587.08
	3	1	1	35.514	2.526	8.377				
	2	2	2	37.081	2.422	8.392				
	4	0	0	43.225	2.091	8.365				
	5	1	1	57.313	1.606	8.346				

**Table 3:** Comparative Analysis of Crystallite Size, Particle Edge Length/Diameter, and Hydrodynamic Diameter of Iron Oxide Nanoparticles (IONPs) with Different Morphologies^a^

morphology	sample name	crystallite size d_c_ (nm)	particle edge length or diameter d_p_ (nm)	Hydrodynamic diameter DLS (nm) (PDI)
cubic	SQ	17.2	31 ± 1	199.7 (0.12)
starlike	ST	21.1	120 ± 1	203 (0.13)
truncated icosahedron	TI	13.7	21 ± 1	197.1 (0.10)
spherical	SP	7.37	11 ± 1	149.4 (0.10)

a This table provides a detailed comparison of the crystallite size (d_c_), measured using X-ray diffraction (XRD) and calculated with the Scherrer equation, the physical particle edge length or diameter (dp), determined through Transmission Electron Microscopy (TEM), and the hydrodynamic diameter (DLS) measured by Dynamic Light Scattering (DLS), across four distinct IONP morphologies. The data highlight the differences between the nanoparticles’ core size and their effective size in suspension, attributed to the oleic acid surface coating and aggregation behavior in aqueous media. The Polydispersity Index (PDI) values are also provided to indicate the size distribution in the suspension.

Rearranging and taking the natural logarithm (ln) of both sides of eq 2 gives
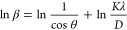
5

Empirical analysis, derived from data compilation (see Table S2 and Figure S3 in Supporting Information), enables the determination of the intercept, c, expressed as
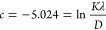
6

Finally, by calculating and replacing the values for *K* and λ into our equation, we find the crystallite size of the starlike *D*_*ST*_:
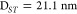
8

The calculated D values were below 30 nm (Table 4). for the four morphologies studied. Consequently, transmission electron microscopy (TEM) plays an essential role in precisely determining particle sizes.

**Table 4:** Summary of the Surface Area Calculations for Nanoparticles of Different Geometries^a^

geometry	formula used	area value (nm^2^)
cubic	A = 6a^2^	5766
starlike	specialized for starlike (SI)	5289
truncated icosahedron	A = 12 × A_pentagon_ + 20 × A _hexagon_	5881
spherical	A = 4πr^2^	380.13

a For cubic and spherical nanoparticles, standard formulas based on edge length (a) and radius (r), respectively, were used. A specialized formula was applied to the starlike nanoparticles, accounting for their unique shape. Truncated icosahedron nanoparticles had their surface areas calculated using a formula specific to their 12-faced structure of pentagon and 20 hexagons, in Supporting Information is detailed. The table presents the calculated area values in square nanometers (nm^2^), highlighting the dodecahedron as having the highest surface area among the geometries studied.

The synthesis of these IONPs can be understood through the LaMer principle, which delineates the stages of nucleation and growth in the formation of monodisperse particles controlling the growth phase where the nanoparticle concentration is maintained below the supersaturation threshold, preventing further nucleation and ensuring uniform particle growth.^40,41^

For ST-IONPs, prolonged growth phases at elevated temperatures, combined with the use of oleic acid in trioctylamine (TOA). In the case of SQ-IONPs, the inclusion of NaCl as a reactant influenced the nanoparticle morphology and size. The shorter reaction time compared to ST-IONPs demonstrated the critical role of reaction duration in controlling nanoparticle growth. SP-IONPs achieved well-defined particles by optimizing the surfactant amount and reaction conditions, effectively separating the nucleation from the growth phase. The use of 1-octadecene as a solvent, combined with oleic acid, provided a stable environment for the formation of uniform nanoparticles.^42^ TI-IONPs are synthesized under a similar solvent to SP-IONPs but with reduced reaction time. Despite changes in reaction conditions, the crystal structure of the nanoparticles remained consistent, indicating robust control over the synthesis process.^43^

**Figure 2:**
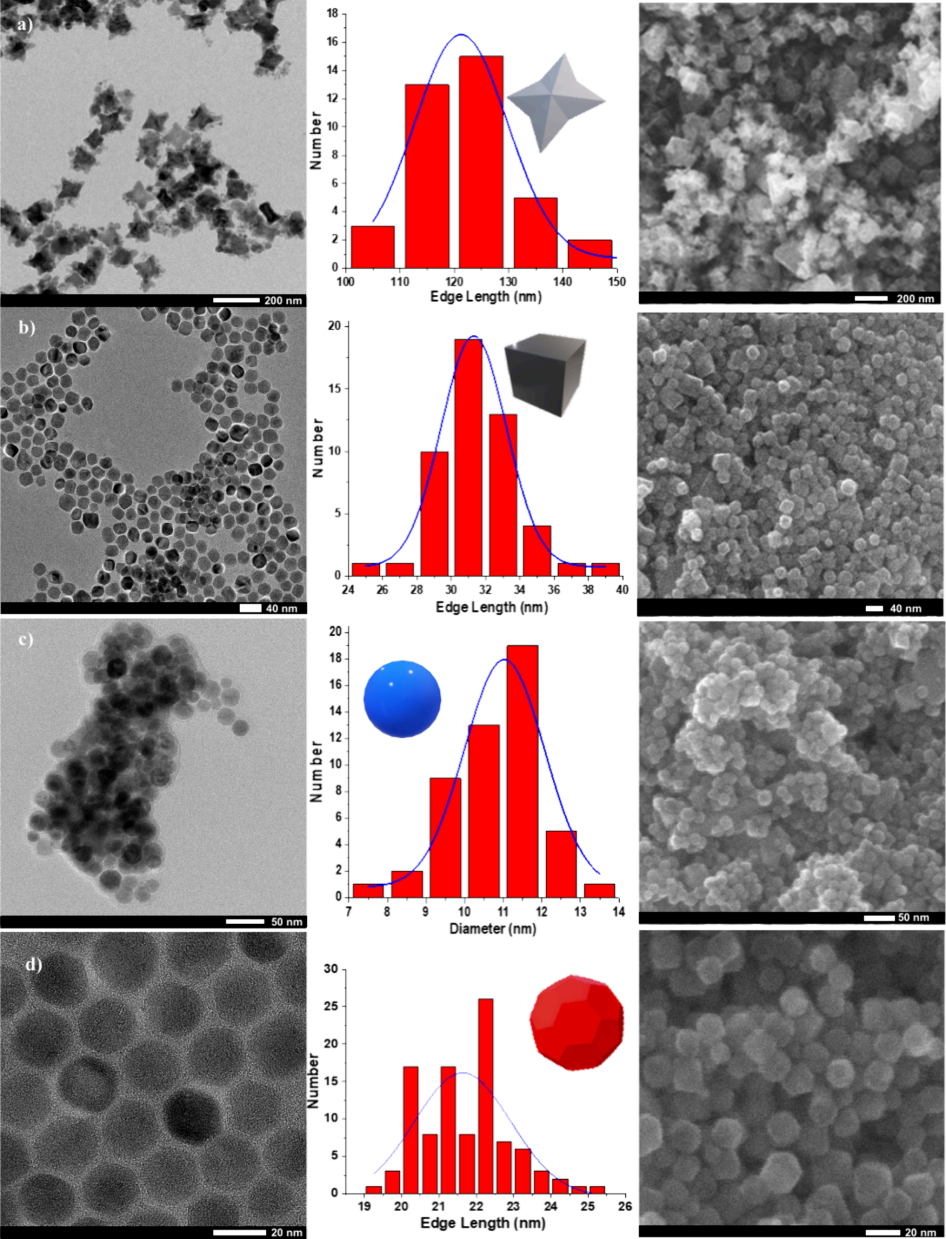
TEM (left), SEM (right) images, histogram, and size distribution of IONPs different geometries: (a) starlike (120 nm), (b) cubic (31 nm), (c) spherical (11 nm), and (d) truncated icosahedron (21 nm).

The synthesized nanoparticles showed a size distribution range of 10 to 150 nm, with well-defined and monodispersed morphologies such as cubic, spherical, four-pointed stars and truncated icosahedron shapes, as observed in the TEM and SEM images (Figure 2). The observed variability in the size and shape of the nanoparticles was attributed to the variation in the organic solvent used, processing temperature, and batch residence time. Notably, the synthesized nanoparticles exhibited a high degree of structural uniformity, which makes them promising candidates for various applications.

IONPs were synthesized with a surface coating of oleic acid to minimize their agglomeration and to impart stability to the nanoparticles. However, when these coated nanoparticles are dispersed in water for Dynamic Light Scattering measurements (DLS), the hydrophobic oleic acid coating on the surface of the nanoparticles still tends to aggregate due to the hydrophilic nature of water. This leads to the formation of clusters of nanoparticles. As a result, hydrodynamic diameters in the range of 149–200 nm are reported (see Table 4). To confirm the presence of iron (Fe) and oxygen (O) in the synthesized nanoparticles, their elemental composition and distribution were analyzed by EDS. The EDS mapping (Figure S4, Supporting Information) revealed that both Fe and O were homogeneously distributed across the surface of the nanoparticles.

The surface areas are calculated for each geometry based on their edge lengths (see Table 5). Cubic nanoparticles exhibited a surface area of 5766 nm^2^, corresponding to an edge length of 31 nm. Starlike nanoparticles require a more nuanced approach to surface area calculation due to their complex geometry, resulting in a surface area of 5289 nm^2^ from an edge length of 120 nm. The truncated icosahedron nanoparticles with an edge length of 21 nm, result in the largest surface area of the IONPs tested here corresponding to value of 5881 nm^2^. Spherical nanoparticles showed a surface area of 380.13 nm^2^, derived from a diameter of 11 nm. These values are further elucidated by comparing the surface areas with the respective edge lengths or diameters The comprehensive comparison underscores that truncated icosahedron nanoparticles have the largest surface area relative to their size, which is particularly relevant for applications that benefit from a large surface-to-volume ratio, such as functionalization and catalysis.

**Table 5:** Specific Assigned Bands Correspond to Vibrational Modes for the Raman Spectra of Iron Oxide Nanoparticles (IONPs)

			band assignment (cm^–1^)				
IONPs							ref
ST	182	342	464	554	665	1091	
	*T*_2*g*_^1^	E_g_	*T*_2*g*_^2^	*T*_2*g*_^3^	A_1g_ spinel crystal structure of Fe_3_O_4_	υ(C–C) gauche from oleic	(45)
SQ	476	563	796	1088		–	
	*T*_2*g*_^1^	*T*_2*g*_^3^	oxidation of Fe(II) to Fe(III) at octahedral sites	υ(C–C) gauche from oleic acid			(46)
SP	215	273	383	1327	1572	–	
	*T*_2*g*_	*T*_1*u*_	vibrations of tetrahedra and octahedra formed by Fe^2+^ and Fe^3+^ atoms	carboxylate stretching	carboxylate stretching		(46, 47)
TI	215	276	391	1301	1601	–	
	*T*_2*g*_	*T*_1*u*_	vibrations of tetrahedra and octahedra formed by Fe^2+^ and Fe^3+^	carboxylate stretching	carboxylate stretching		(46)

### Composition

Raman spectroscopy is a valuable tool for elucidating the composition of IONPs and establishing meaningful correlations with their magnetic properties (Figure S1, Supporting Information). By analyzing distinctive vibrational modes exhibited by each IONP, considering their respective diameters, we successfully assigned specific peaks observed in the Raman spectra. The peak assignments were made by comparing the experimental data with established literature references for magnetite (Table 5). Notably, discernible shifts in the wavenumbers assigned to T_2g_ vibrational modes were observed across the various IONPs. It is well-documented that wavenumber shifts in the Raman spectra of nanoparticles exhibit an inverse relationship with the nanoparticle diameter.^44^ Our findings indicate that magnetite predominates as the iron oxide species in all the examined IONPs.

ATR-FTIR spectroscopy was employed as a precise analytical technique to elucidate the distinctive Fe–O vibration modes of magnetite, as well as other normal modes, as illustrated in (Figure S2, Supporting Information). The spectral analysis revealed prominent absorption bands centered around 560 cm^–1^, which can be attributed to the characteristic Fe–O vibrations of magnetite. Moreover, in the SQ sample, additional bands corresponding to C–C and C–O vibrations were observed within the spectral regions of 880 cm^–1^ to 1018 and 1360 cm^–1^ to 1456 cm^–1^, respectively.^48,49^ Several higher frequency peaks in the vibrational spectra, i.e. 2437 cm^–1^(Raman) and 2880, 2928 cm^–1^ (FT-IR), are observed for the SQ geometry. We have not been able to clearly assign these peaks, but believe they are due to side reactions from the starting materials that do not affect the magnetic properties of the IONPs.

### Magnetic Properties

**Figure 3:**
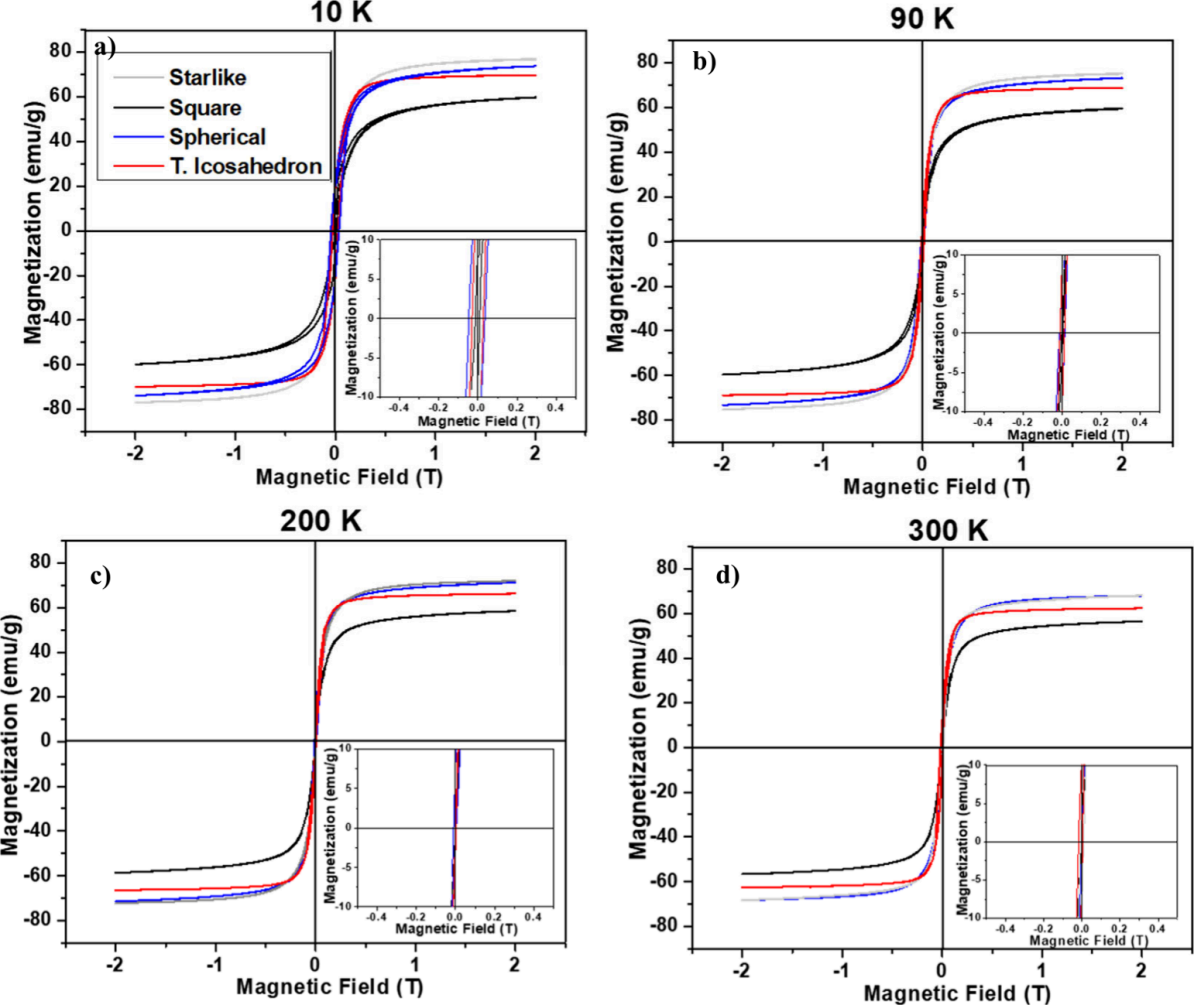
Magnetic Hysteresis Curves of Iron Oxide Nanoparticles (IONPs) at Various Temperatures. The figure displays the magnetic hysteresis curves of the iron oxide nanoparticles measured using a vibrating sample magnetometer (VSM) at different temperatures, including (a) 10 K, (b) 90 K, (c) 200 K, and (d) 300 K. The magnification of the saturation region allows for a closer examination of the magnetic properties of the nanoparticles.

The magnetic properties of the synthesized nanomaterials are influenced by a combination of factors: size, surface area, geometry, and crystallographic phase. These factors can be analyzed through the relationship between temperature and magnetic field dependence using techniques such as Vibrating Sample Magnetometry (VSM) and Physical Property Measurement System (PPMS). The magnetic properties of the synthesized nanoparticles were investigated using hysteresis curves measured with VSM at temperatures of 10, 90, 200, and 300 K, as shown in Figure 3. The saturation magnetization (M_s_), remanence (M_r_), and coercivity (H_c_) were measured at each of these temperatures for the four different samples. The obtained results are presented in Table 6.

**Table 6:** Measurements of Saturation Magnetization (Ms), Remanence (Mr), and Coercivity (Hc) for Four Types of Iron Oxide Nanoparticles at Four Different Temperatures

	temp											
	10 K			90 K			200 K			300 K		
sample name	Ms (emu/g)	*M*_r_ (emu/g)	Hc (Oe)	Ms (emu/g)	*M*_r_ (emu/g)	Hc (Oe)	Ms (emu/g)	*M*_r_ (emu/g)	Hc (Oe)	Ms (emu/g)	*M*_r_ (emu/g)	Hc (Oe)
SQ	59.9	10	128	59.4	11.9	28.3	58.54	0.334	7.04	56.67	45.8	54
ST	73.8	21.29	460	75.19	5.25	63	72	8.2	100	68.24	5.8	74
TI	70.2	19.04	290	69	9.65	115	66.3	1.1	9.74	62.4	11.4	176
SP	77.2	15.7	250	73	1.096	123	71.37	1.25	15.6	68	6.2	4.6

**Figure 4:**
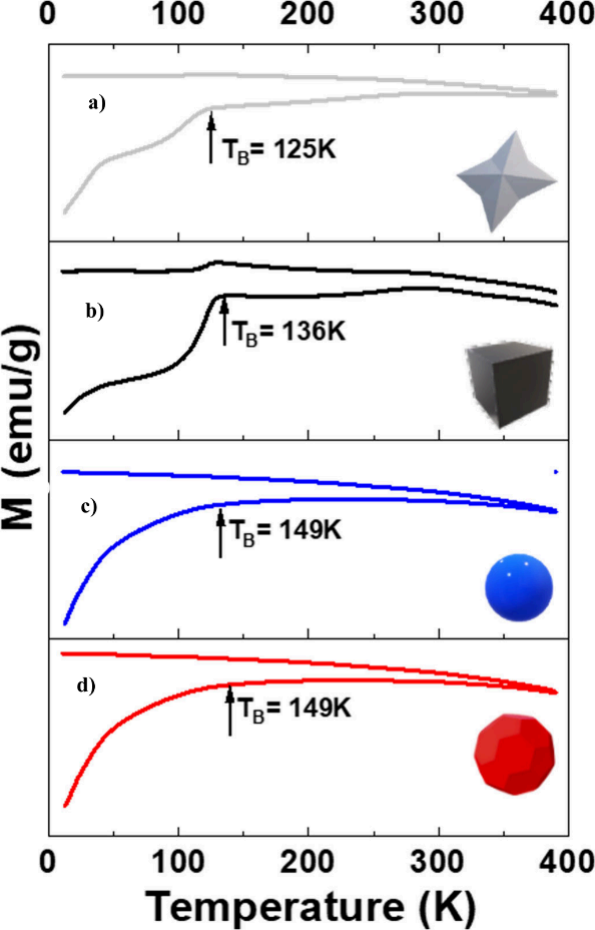
Temperature-dependent magnetization (M-T) curves were obtained by ZFC/FC (500 Oe) measurements using a physical property measurement system (PPMS) to show the blocking temperature (Tb): (a) starlike (125 K), (b) cubic (136 K), (c) spherical (149 K) and (d) truncated icosahedron (149 K).

The hysteresis loops of the IONPs exhibit superparamagnetic behavior in accordance with the saturation law, i.e. when an external magnetic field is applied to a material, its magnetic moment increases linearly with the field strength until it reaches a saturation point. At this stage, the magnetic moment becomes independent of the magnetic field strength and is determined by the intrinsic properties of the material,^50^ indicating if the nanoparticle’s anisotropy is comparable to the thermal energy and magnetization. This behavior is typical of superparamagnetic materials, which are characterized by a lack of magnetic remanence and coercivity at temperatures above the blocking temperature.^51,52^ The saturation magnetization (M_s_) values of the synthesized IONPs show a positive correlation with their edge length or diameter (d_p_) for all samples. The trend is consistent with the superparamagnetic behavior where larger nanoparticles exhibit higher magnetization due to their greater magnetic moments.^53^ At 300 K, star-shaped and spherical IONPs exhibit high magnetization saturation (68.24 emu/g and 68 emu/g, respectively). Interesting anomalies were observed in the case of the spherical nanoparticles, which have a smaller size, similar magnetization (M_s_) and low coercivity (H_c_) compared to the star-shaped ones. The (M_s_) and low (M_r_) and (H_c_) values in the spherical case may be attributed to multiple spin orientations in the spherical structure, leading to agglomeration contrary to the truncated icosahedron geometry at 300 K. This effect can be caused by strong spin interactions in highly crystalline structures during the spin alignment.^54^ In paramagnetic or superparamagnetic nanoparticles, a low coercivity indicates that the material is more susceptible to external magnetic fields and can be easily manipulated or controlled.^55^ At low temperatures (10 K), the thermal energy is reduced, and magnetic domains are more stable. This can lead to higher coercivity values. ST, SP, and TI nanoparticles show a coercivity of 250 to 470 Oe, indicating that the material is in a ferromagnetic state at this temperature, as such a value is significantly higher than what would be expected for paramagnetic materials.^56^ The blocking temperature (*T*_*B*_) is a characteristic temperature of IONPs at which the thermal energy is large enough to overcome the anisotropy energy barrier of the magnetic moment. This results in a reduction of the magnetic moment and a decrease in coercivity, leading to a superparamagnetic state.^57^ Obtained from the Zero Field Cooling (ZFC) and Field Cooling (FC) graph (see Figure 4), the truncated icosahedron and spherical type show a *T*_*B*_≈ 149 K, while in the case of starlike *T*_*B*_≈ 125 K and cubic the *T*_*B*_≈ 136 K. This difference in *T*_*B*_ values can be attributed to several factors, such as the shape anisotropy, size distribution, and surface effects of the nanoparticles. In the case of spherical and truncated icosahedron nanoparticles, their high *T*_*B*_ values may be due to their larger size and more uniform size distribution, which can reduce thermal fluctuations and enhance magnetic stability. Additionally, their crystallographic planes may also contribute to their high *T*_*B*_ values by providing a higher energy barrier for magnetic moment rotation. The cubic and starlike nanoparticles may have a lower *T*_*B*_ value due to their more irregular shape, which can lead to a greater variation in size and shape anisotropy, resulting in a weaker magnetic moment coupling and lower thermal stability. However, the exact mechanism behind the differences in *T*_*B*_ values between different morphologies is still an active area of research and may vary depending on the specific nanoparticle system under study.^58,59^

### MR Relaxation Properties

**Figure 5:**
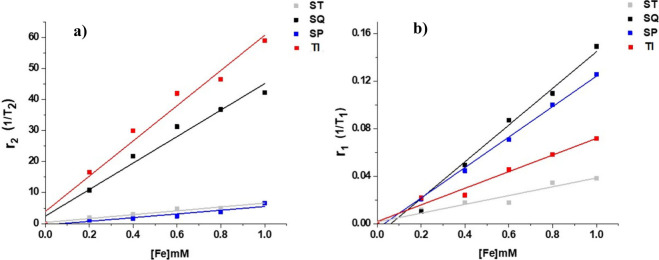
Magnetic Resonance (MR) relaxivities measurements. (a) The plot displays the relationship between the transverse relaxation rate (r_2_) and the concentration of iron [Fe] in deionized water. The r_2_ values were obtained through MR relaxivity measurements, which reveal the efficacy of iron oxide nanoparticles as T_2_ contrast agents. (b) The plot shows the relationship between the longitudinal relaxation rate (r_1_) and the concentration of iron [Fe] in deionized water. The r_1_ values were measured using magnetic resonance (MR) relaxivity measurements, which provide information about the efficiency of iron oxide nanoparticles as T_1_ contrast agents.

The size, morphology and shape of IONPs play a critical role in their relaxivity properties. In general, larger particles have a greater magnetic moment and produce a stronger magnetic field perturbation. However, larger particles tend to aggregate, reducing their effectiveness as contrast agents.^60^ In this project, the feasibility of using these IONPs coated with oleic acid as T_1_ and T_2_ contrast agents has been investigated. The relaxivities were determined using a specific relation that relates the relaxation rate to the concentration of the contrast agent.^61^ The relaxivities provide information about the efficiency of the nanoparticles as contrast agents in modulating the relaxation times of water molecules. These relaxivities were calculated based on experimental measurements of relaxation times at different concentrations of the iron oxide nanoparticles and are expressed as longitudinal (T_1_) and transverse relaxation (T_2_) by the relationship (eq 7)^62^
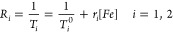
7where  and *r*_*i*_ represent the relaxation rates of protons in the absence of nanoparticles and [*Fe*] correspond to the analytical concentration of IONPs.^63^ The diffusion of water molecules near magnetized IONPs causes nuclear magnetic relaxation, which is replicated by chemical exchanges. As a preliminary study of using IONPs coated by oleic acid as a contrast agent, the proton relaxation time in a range of concentrations (0–1.0 mM) was measured (see Figure 5). Iron oxide nanoparticles’ truncated icosahedron and cubic geometries exhibited significantly higher transverse relaxation time (r_2_) responses than the star type and spherical geometries (see Table 7). The truncated icosahedron nanoparticles showed an r_2_= 56.77 s^–1^mM^–1^ while the cubic nanoparticles had an r_2_ = 42.67 s^–1^mM^–1^ with a linear dependency of transverse relaxation yields. In contrast, the star type and spherical nanoparticles had lower r_2_ values of 6.22 s^–1^mM^–1^ and 5.96 s^–1^mM^–1^, respectively. The r_2_/r_1_ ratio, which is an indicator of the suitability of a contrast agent for T_2_-weighted imaging.,^64^ was significantly higher for the TI and cubic geometries compared to the star type and spherical geometries. The TI nanoparticles had an r_2_/r_1_ ratio of 760, while the cubic nanoparticles had an r_2_/r_1_ ratio of 277. On the other hand, the star type and spherical nanoparticles had lower r_2_/r_1_ ratios of 167 and 78. The higher saturation magnetization impacts the spin–spin relaxation, influencing the surrounding water molecules’ transverse relaxation time (T_2_).^65^

**Table 7:** Comparison of r_2_, r_1_, r_2_/r_1,_ and Regression Values

sample	r_2_ (s^–1^ mM^–1^)	R^2^	r_1_ (s^–1^ mM^–1^)	R^2^	r_2_/r_1_
TI	56.77 ± 4.4	0.99	0.07 ± 0.01	0.99	760
SQ	42.67 ± 3.26	0.99	0.154 ± 0.01	0.99	277
ST	6.22 ± 0.58	0.96	0.037 ± 0.01	0.97	167
SP	5.96 ± 0.9	0.96	0.076 ± 0.01	0.93	78

## Conclusions

This study addresses the synthesis and characterization of iron oxide nanoparticles (IONPs) with various morphologies and their potential applications as MRI contrast agents. The results demonstrate the successful formation of magnetite phase nanoparticles with well-defined crystallographic structures and high purity. The X-ray diffraction (XRD) analysis confirmed the spinel crystal structure of magnetite, and the Scherrer equation provided information about the average crystallite size between 13 and 20 nm for different morphologies. The nanoparticles exhibited a wide range of sizes and shapes, including cubic, spherical, four-pointed stars, and truncated icosahedron structures, with size distributions of the nanoparticles in the range of 10 to 150 nm. The variation in morphology was attributed to the different solvents used, processing temperature, and batch residence time. Importantly, all the synthesized nanoparticles showed a high degree of structural uniformity, making them promising candidates for various biomedical applications. The synthesis process for these iron oxide nanoparticles systems has been validated through multiple iterations. We have developed and standardized an optimization protocol that reliably produces nanoparticles with the specific geometries described in our study. The nanoparticles were coated with oleic acid to prevent agglomeration and provide stability. However, when dispersed in water, the hydrophobic coating tended to aggregate, forming nanoparticle clusters. The hydrodynamic diameter measurements confirmed the presence of nanoparticle clusters with hydrodynamic diameters between 149 and 200 nm in water. The Raman spectroscopy analysis confirmed the presence of magnetite as the predominant iron oxide species in all the synthesized IONPs. The distinctive vibrational modes observed in the Raman vibrational modes corresponding to magnetite were observed. The magnetic characterization revealed that the synthesized nanoparticles exhibited superparamagnetic behavior, with saturation magnetization (Ms) values positively correlated with their diameter. The nanoparticles showed low remanence (Mr) and coercivity (Hc) values, indicating their susceptibility to external magnetic fields and ease of manipulation. The T_B_ measurements indicated the superparamagnetic state of the nanoparticles, with variations depending on the morphology, size distribution, and surface effects. Overall, the synthesized IONPs demonstrated desirable physicochemical properties for potential applications in nanomedicine, including MRI contrast agents. Further studies are needed to explore their toxicity and their performance as drug delivery systems, and other biomedical applications. Additionally, efforts should be directed toward addressing the challenges associated with nanoparticle aggregation in aqueous environments and optimizing their stability and biocompatibility. The truncated icosahedron nanoparticles have stronger paramagnetic behavior, which leads to transverse T_2_ relaxation, compared to other geometries. Additionally, they exhibit superior magnetic and substantial surface area of these nanoparticles, at 5881 nm^2^ behavior, and are well-suited for future biomedical applications. This study delves into the synthesis, crystallographic phases, morphology, optical properties, and potential uses of these nanoparticles. Future studies will focus on exploring alternative coatings for iron oxide nanoparticles (IONPs) to improve their aqueous dispersion and stability.

## Supporting Information Available

Materials, instrumentation, ICP analysis results, Raman and ATR-FTIR spectra, parameters obtained for modified Sherrer equation, full width at half-maximum, theta values and beta considering the crystallographic peak positions, EDS mapping and surface area calculation for all geometries (PDF)
