# Supplementary material for: Geometry and Surface Area Optimization in Iron Oxide Nanoparticles for Enhanced Magnetic Properties
Source: ACS Omega. 2024 Jul 18;9(30):32980–90. doi: 10.1021/acsomega.4c03988 (PMC11292628; doi:10.1021/acsomega.4c03988)
Supplement: Supplementary file 1 — ao4c03988_si_001.pdf [file ao4c03988_si_001.pdf]

Supplementary Information

Geometry and Surface Area Optimization in Iron Oxide Nanoparticles for Enhanced Magnetic Properties

Alexis Lavín Flores,[*†,ǂ] Nataniel Medina-Berríos,[†,ǂ] Wenndy Pantoja-Romero,[†,ǂ] Dariana Berríos Plaza[µ], Kim Kisslinger[α], [β]Juan Beltran-Huarac, Gerardo Morell[†,‡] and [†,ǂ]Brad R. Weiner

[†] Molecular Sciences Research Center, University of Puerto Rico, San Juan, PR 00926-2614, USA.

[ǂ] Department of Chemistry, University of Puerto Rico, Río Piedras Campus, San Juan, PR 00925-2537, USA.

[‡] Department of Physics, University of Puerto Rico, Río Piedras Campus, San Juan, PR 00925-2537, USA.

[µ]Department of Biology, College of Natural Sciences, University of Puerto Rico, Rio Piedras Campus, San Juan, PR 00925-2537, USA.

[α]Center for Functional Nanomaterials, Brookhaven National Laboratory, Upton, NY-11973, USA.

[β]Department of Physics, Howell Science Complex, East Carolina University, Greenville, NC 27858, USA.

[*]Corresponding author: alexis.lavin@upr.edu

Materials details

In the preparation of various morphologies of iron oxide nanoparticles, high-purity chemicals were utilized to ensure reproducibility and accuracy in the synthesis processes. Iron(III) chloride hexahydrate ($FeCl_3 \cdot 6H_2O$, 98%) and sodium oleate (97%) were procured from Sigma Aldrich and Tokyo Chemical Industry (TCI), respectively. The solvent employed across the syntheses (ethanol, hexane and isopropanol (99%) ACS grade), was obtained from Sigma Aldrich, Oleic acid (NF/FCC grade) and tri-n-octylamine (TOA, 97%) were sourced from ACROS Organics and Fisher Chemical, serving as the surfactant and high-boiling organic solvent, respectively, critical for the nanoparticle formation and stabilization. Sodium chloride (NaCl, 98%), used in the cubic nanoparticle synthesis to influence particle morphology, was also supplied by Sigma Aldrich. All reactions were conducted using nanopure water, produced by Milli-Q® EQ 7000 Ultrapure Water Purification System to obtain ultrapure water quality [resistivity 18.2 MΩ·cm @ 25 °C; total organic carbon (TOC) ≤ 5 ppb] to avoid any impurities that could interfere with the nanoparticle synthesis.

Instrumental Section

The crystallinity phases of the synthesized nanomaterials were analyzed by a Rigaku XtaLAB SuperNova X-ray diffractometer with micro-focus Cu-Kα radiation (λ=1.5417 Å) source. The diffractometer was equipped with a HyPix3000 X-ray detector. Experiments were carried out in transmission mode operating at 50 kV and 1 mA and the samples were mounted in MiTeGen micro-loops.

High-resolution analytical scanning/ transmission electron microscope (S/TEM) images, energy dispersive X-ray spectrometer (EDS) for elemental and compositional mapping, and selected area diffraction (SAED) data were obtained using an FEI Talos F200X HR-TEM and JEOL JEM-2100F transmission electron microscope (JEOL Ltd.) operating at 200 kV. To prepare the TEM

grid sample, 25 µL of diluted oleic acid-coated nanoparticles were mounted on 'carbon film only on 200 mesh, Copper' grid (Ted Pella, Inc.), and allowed to dry overnight.

Raman spectra were performed using a Thermo Scientific™ DXR3 Raman Microscope with a 532nm excitation laser. The laser power was adjusted to prevent magnetite phase transformations.

FTIR spectra were obtained with a Bruker Tensor 27 with diamond ATR in the range of 400-4000 cm^{-1} and the samples were mounted on microscope slides.

The relaxivities measuremennts longitudinal and transverse (r1 and r2) were obtained using a NMReady-60PRO benchtop relaxometer (Nanalysis Corp. Canada). The concentration of Fe ([Fe]) of 0, 0.2, 0.4, 0.6, 0.8 and 1.0 mM was maintained in nanopure water and scanned applying 1.41 T at 30 °C to obtain the longitudinal relaxation time (T1) and transverse relaxation time (T2) values.

The magnetic field M(H) and temperature dependent M(T) magnetic measurements, were measured in te equipment based on physical property measurement system (PPMS) DynaCool (Quantum Design, Inc.) using the vibrating sample magnetometry (VSM) technique. The powder sample (1–10 mg) was dispossed in VSM powder sample holder (part no. 4096-388, Quantum Design, Inc.).

The quantification of [Fe] in all samples was obtained from inductively coupled plasma-optical emission spectrometry (ICP-OES)(Table S 1), the samples was treated using acid digestion (3% HCl) technique. The Calibrations were performed using ionic Fe standards in HCl from Iron AA Standard (1000 ppm of Fe in 3% HCl) ranging from 0 to 100 parts per million (ppm). The Fe content of the resulting solutions was measured by PerkinElmer Optima 8000 Optical Emission Spectrometer using ICP continuous technique obtaining the data of Table S 1.

Table S 1. Iron (Fe) Concentration in Various Samples Determined by ICP Analysis. This table includes five trials for each sample (ST, SQ, SP, HX), showing the Fe concentration in ppm, the standard deviation of these trials in ppm, and the average concentration in mM according to digested solution.

Sample	Trial N°	ICP results [Fe] (ppm)	Mean	Std. Dev (σ)	[Fe] (mM)
ST	1	21.59	21.57	0.35	386.24
	2	21.27			
	3	21.66			
	4	22.1			
	5	21.22			
SQ	1	27.68	28.02	0.37	501.72
	2	28.59			
	3	28.18			
	4	27.57			
	5	28.07			
SP	1	31.78	31.58	0.41	565.45
	2	31.78			
	3	32.13			
	4	31.05			
	5	31.15			
TI	1	25.52	25.44	0.29	455.58
	2	25.29			
	3	25.96			

	4	25.35			
	5	25.09			

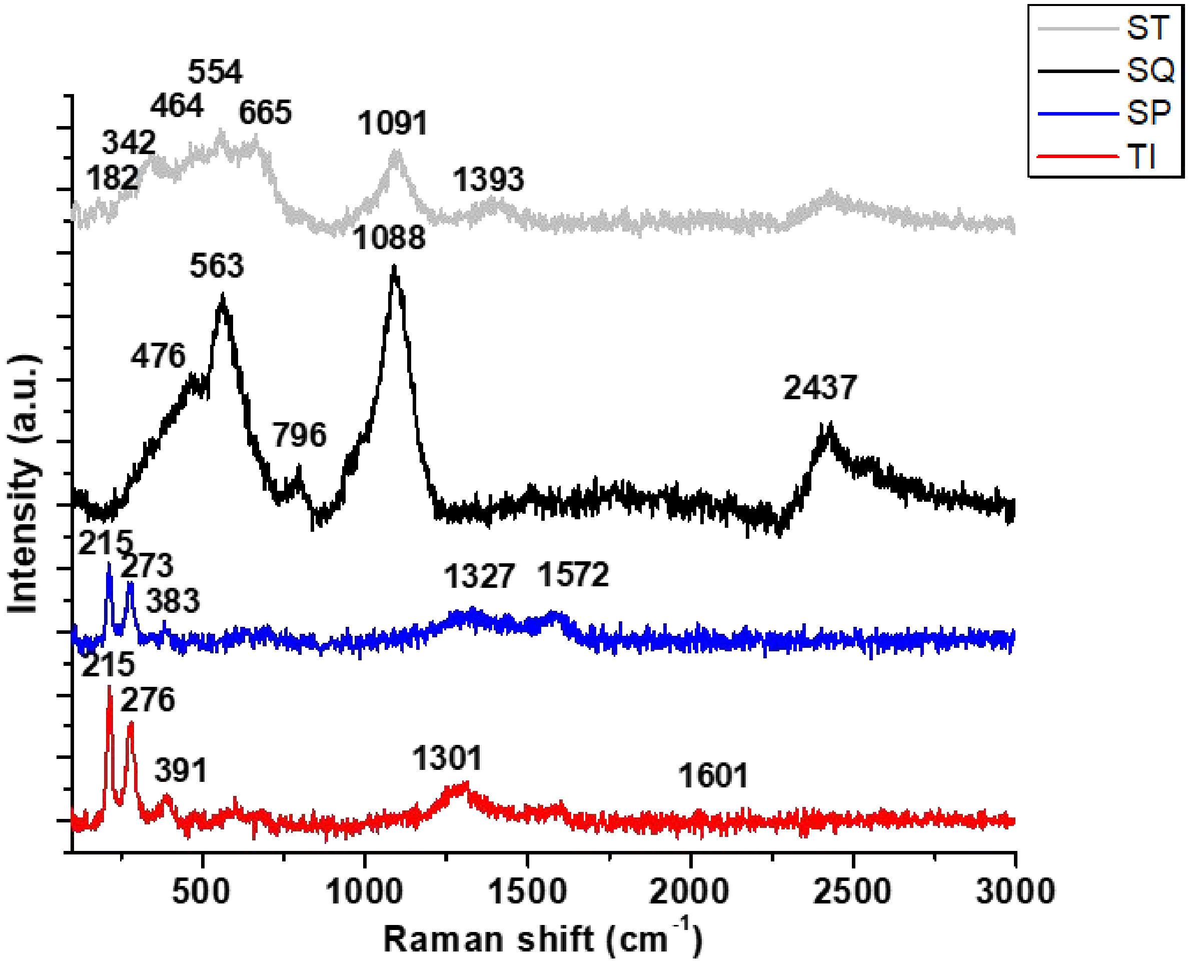

Figure S 1. Stacked Raman spectra of the IONPs

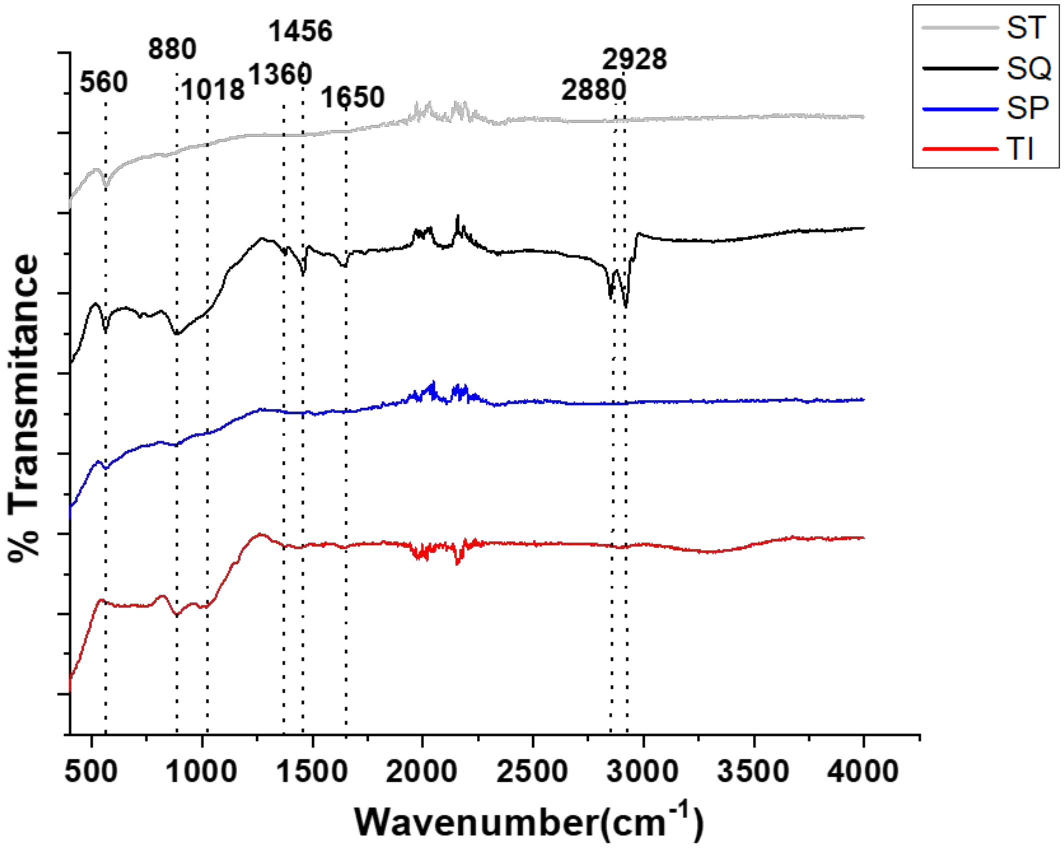

Figure S 2. Attenuated Total Reflection Fourier Transform Infrared Spectroscopy (ATR/FTIR) analysis conducted on the iron oxide nanoparticles (IONPs*)*.

Table S 2. Full width at half maximum, theta values and beta considering the crystallographic peak positions.

Signal.No	Peak position	FWHM	ln(1/cosθ)	ln(β)
1	**35.55**	0.5198	0.0489144	-4.70254
2	**30.1**	0.4652	0.0349025	-4.81351
3	**37.12**	0.2664	0.0534104	-5.37098
4	**43.155**	0.4548	0.0726557	-4.83612
5	**57.069**	0.503	0.1295056	-4.73539

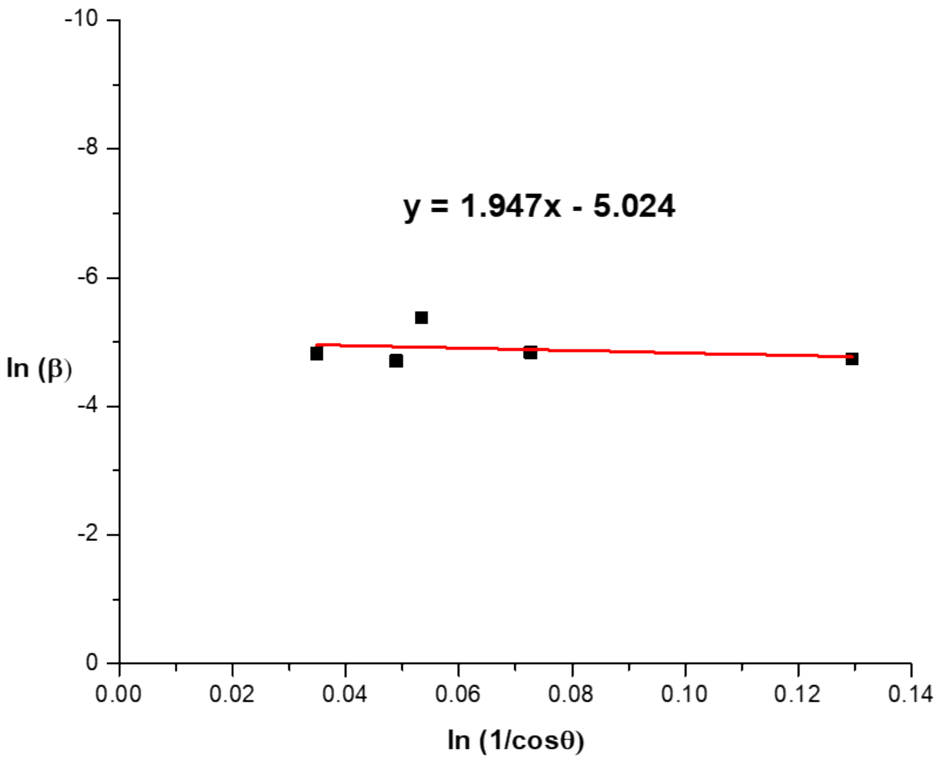

Figure S 3. Graphical Analysis of Modified Scherrer Equation. The figure illustrates the linear correlations derived from the modified Scherrer equation, plotting ln(β) against ln(1/cosθ).

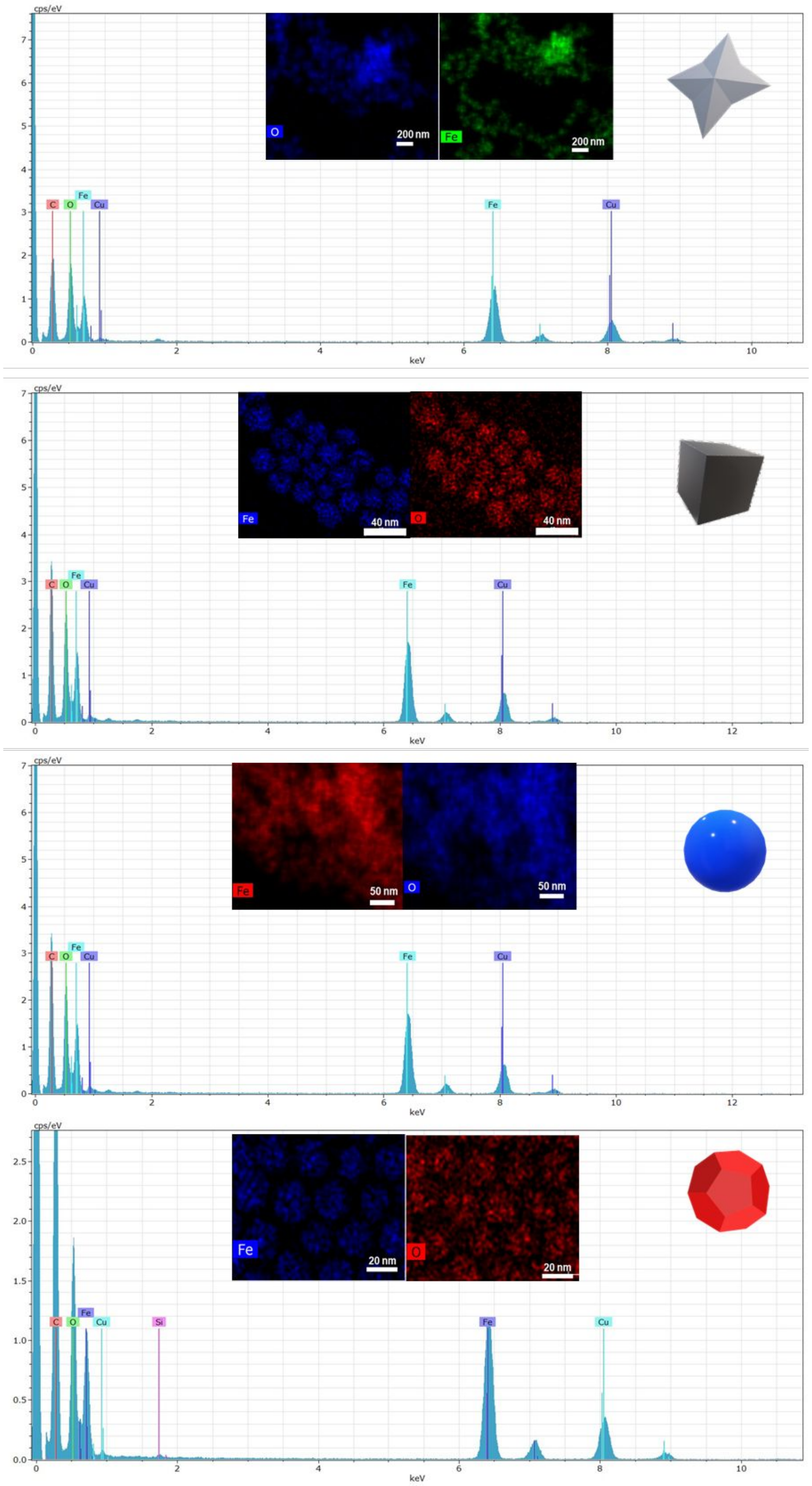

Figure S4. EDS mapping of starlike, cubic, spherical, and hexagonal geometries, illustrating the elemental distribution.

Calculations for surface area

4-pointed Star-like

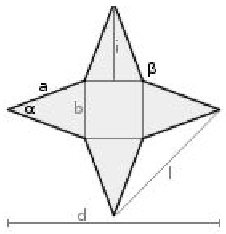

$$\beta = 90° + \alpha$$

$$b = \sqrt{2 * a^2 * (1 - \cos(\alpha))}$$

$$i = \sqrt{(4 * a^2 - b^2) / 4}$$

$$l = \sqrt{2 * a^2 * (1 - \cos(\beta))}$$

$$p = 8 * a$$

$$A = 2 * i * b + b^2$$

Where; a=45, inner angle (α)=45°, outer angle (β)=150°, Area = 5530 nm^2

Truncated Icosahedron.

Pentagon Area Formula:

The area of a regular pentagon with side length **s** can be calculated using the formula:

$$A_{pentagon} = \frac{1}{4}\sqrt{5(5+2\sqrt{5})}s^2$$

This formula is derived from geometric principles involving the pentagon's properties, specifically using the Golden Ratio in the context of a pentagram.

Hexagon Area Formula:

The area of a regular hexagon with side length **s** is given by:

$$A_{hexagon} = \frac{3\sqrt{3}s^2}{2}$$

This formula is standard for a regular hexagon, derived from combining six equilateral triangles.

Using these formulas, the total surface area of a truncated icosahedron (which consists of 12 pentagons and 20 hexagons) is calculated by:

$$A_{total} = 12\ x\ A_{pentagon} + 20\ x\ A_{hexagon}$$

Considering the side length as 8.1 nm, measured from the TEM images.
